# Deception Detection in Action: Embodied Simulation in Antisocial Human Interactions

**DOI:** 10.3389/fpsyg.2017.00166

**Published:** 2017-02-07

**Authors:** Rouwen Cañal-Bruland

**Affiliations:** Department of Sport Psychology, Institute of Sport Science, Friedrich-Schiller-University JenaJena, Germany

**Keywords:** action, perception and action, cognition, emotion, embodiment, deception detection, social interaction, embodied simulation

Spotting the intentions of a pickpocket in a crowded environment may save a few dollars. If you are a police officer, then identifying a suspect who is pretending to reach for a wallet while actually pulling a gun can be a matter of life or death. These examples illustrate that detecting deceptive intentions from other persons' actions is of great practical importance in many social contexts. Although it is well known that humans can identify deceptive intentions based on bodily cues, our understanding of deception detection, however, is still quite limited, partly because a comprehensive theoretical framework of deception detection is lacking. This is different for pro-social human interactions like playing a piano duet. In this context, overarching and unifying explanations are available based on the concept of embodied simulation. Here I propose that embodied simulation is perhaps the most promising steppingstone to develop a comprehensive embodied theory of deception detection as well. Embodied simulation is typically construed as the interplay between action and perception and it also incorporates the interplay with cognition and emotion. In my view, integrating and studying motor, perceptual, cognitive and emotional processes is imperative to understand how deceptive intentions can be detected from human movements. This opinion paper aims at fleshing out this idea and providing some first suggestions and hypotheses on how to achieve the ultimate goal to develop an encompassing embodied theory of deception detection in (anti)social interactions.

To start with, deception can be defined as an act that aims to mislead an observer into making an incorrect judgment about the actor's (i.e., deceiver's) true action intention (Cañal-Bruland and Schmidt, 2009). To date most research on deception has been dedicated to detecting deceit in verbal communication (i.e., lying) (e.g., Bond et al., 1992; Frank and Ekman, 1997; Ekman et al., 1999); less research has been devoted to non-verbal cues in catching liars (e.g., Ekman and Friesen, 1969; Vrij, 2004, 2006); and, despite its obvious relevance, remarkably little research has been done to better understand the detection of deceptive intentions embodied in movements (e.g., Runeson and Frykholm, 1983; Cañal-Bruland and Schmidt, 2009; Sebanz and Shiffrar, 2009).

Since the pioneering work by Runeson and Frykholm (1983) it is commonly accepted that observers can distinguish between deceptive and non-deceptive intentions based on bodily cues. Their original study revealed that information conveyed by joint kinematics alone was not only sufficient to accurately judge the weight of a carried box but also to recognize whether the actor carrying the box intended to mislead the observer about the true weight of the box. Yet, despite Runeson and Frykholm's important finding, as well as recent work exploring expertise effects and neuronal mechanisms contributing to successful deception detection from bodily actions (e.g., Kunde et al., 2011; Brault et al., 2012; Tomeo et al., 2012; Mori and Shimada, 2013; Wright et al., 2013; Renden et al., 2014; Wright and Jackson, 2014), the underlying psychological processes that allow human observers to successfully recognize deceptive intentions from others' bodily actions remain largely unknown.

To unravel the psychological processes that allow human observers to successfully recognize deceptive actions from others' bodily actions, it is of paramount importance to develop and establish an encompassing theoretical framework to study and explain deception detection. A novel theoretical backdrop is necessary because in contrast to the study of pro-social human interactions (including joint actions such as playing a piano duet, see Sebanz et al., 2006), there is a definite need for such a theoretical framework to account for antisocial human interactions, in which human agents attempt to mislead and potentially harm others. This is particularly true for the field of deception detection.

Here it is proposed that the most promising way to develop a theory for deception detection is to start from recent theories of embodiment that have proven successful in explaining pro-social human interactions by focusing on the tight links between perception and action such as the common coding theory (Prinz, 1997; Hommel et al., 2001; Schütz-Bosbach and Prinz, 2007). These theories are groundbreaking in that they break away from the Cartesian view that perception and action constitute distinct entities. On the contrary, they argue that perceptual processes are grounded in the motor system in a way that action capabilities and intentions shape what we see and how we interpret what we see.

However, these ideas need be extended in order to establish an embodied theory of deception detection, because next to perceptual and motor influences on deception detection, other psychological processes may also contribute to antisocial human interactions such as cognitions (e.g., thoughts) and emotions (e.g., empathic feelings). Hence, I suggest to supplement existing perception-action theories with findings from social cognitive neuroscience pointing at the links between perception and action representations with cognitions and emotions (Gallese, 2001, 2003; Keysers and Gazzola, 2009). Key assumption of this idea is that the ability underlying the successful detection of deceptive action intentions in others' movements is rooted in embodied simulation (Freedberg and Gallese, 2007). Embodied simulation enables observers to (implicitly) activate their own internal representations of bodily states associated with the observed actions, emotions and cognitions (Freedberg and Gallese, 2007). I suggest that a deeper understanding of antisocial human interactions such as in deception detection can only be gained if we integrate not only the mapping of motor and perceptual representations, but also include the interplay between internal representations of bodily states with cognitions and emotions.

The concept of embodied simulation can unify motor, perceptual, cognitive and emotional processes into one integrative framework that has the potential to develop into a novel, comprehensive embodied theoretical framework for the exploration of deception detection (see Figure 1).

**Figure 1:**
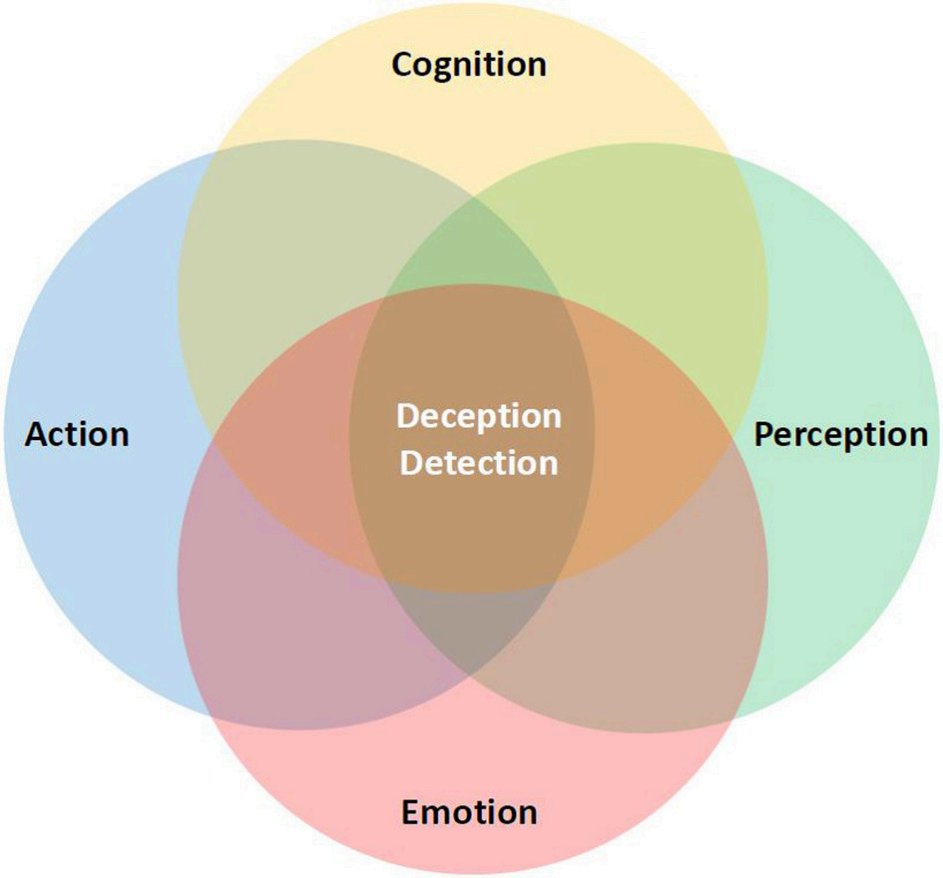
**An embodied theoretical framework for the exploration of deception detection**.

How can one test the assumptions and implications of this view? To start with, common-coding theory argues that the perception and production of (deceptive and non-deceptive) actions share common representations (Prinz, 1997; Hommel et al., 2001; Schütz-Bosbach and Prinz, 2007). Because perception and action are deemed tightly coupled by common representations, they can mutually induce each other. This reciprocal influence has been substantiated by neurophysiological studies that identified brain networks that respond to the observation as well as the production of an action (Di Pellegrino et al., 1992; Rizzolatti and Craighero, 2004). It follows that a high similarity between perceptual and motor representations promotes action perception. That is, observers are predicted to be perceptually better attuned to observed movements that form part of one's own motor repertoire, typically acquired through personal action experience (Schütz-Bosbach and Prinz, 2007). In other words, the more action experience an observer has accumulated with executing a specific movement, the better the perception of the same movement should be when executed by another person (referred to as the “motor experience hypothesis,” Cañal-Bruland et al., 2010). This contention has received empirical support from both behavioral studies (Knoblich and Flach, 2001; Loula et al., 2005; Casile and Giese, 2006; Cañal-Bruland et al., 2011, 2012) and brain imaging recordings (Calvo-Merino et al., 2005, 2006).

There is initial evidence that this hypothesis also holds true for deception detection. Sebanz and Shiffrar (2009) reported that expert basketball players outperformed novices in identifying fake pass movements when presented with short video clips but not with static images (see also Jackson et al., 2006; Cañal-Bruland and Schmidt, 2009; Cañal-Bruland et al., 2010). They also showed that experts maintained their superior performance when presented with point-light-animations (only displaying kinematic landmarks), while novices did not perform better than chance (see also Williams et al., 2009). These results indicate that the motor repertoire of an observer indeed plays a crucial role in deception detection.

As depicted in Figure 1, detecting deception from bodily cues by means of embodied simulation is not exclusively confined to the fundamental links between perception and action but also includes the inference of cognitive and emotional states. There is initial evidence for such interactions showing that knowledge about the likelihood of deceptive actions biases judgments about whether an observed movement may be deceptive or not (Cañal-Bruland and Schmidt, 2009; see also Wright et al., 2013; Mann et al., 2014).

Movements convey critical information (Johansson, 1973, 1976; for a review, see Blake and Shiffrar, 2007) that allow human observers to discriminate subtle movement differences (Cutting et al., 1978; Runeson and Frykholm, 1983; Bertenthal et al., 1985). A seminal experimental approach is to present participants with a subset of anatomical landmarks representing different joint centers of an actor as moving points of light, called point-light displays (PLDs; see Cañal-Bruland and Williams, 2010). Of particular relevance for embodied simulation is that based on such PLDs humans are able to recognize one's own identity (Beardsworth and Buckner, 1981; Jokisch et al., 2006), the identity of friends (Cutting and Kozlowski, 1977), gender (Pollick et al., 2005), and also to predict action intentions and movement effects (Abernethy et al., 2001; Huys et al., 2009; Williams et al., 2009), including the detection of deceptive action intentions (Runeson and Frykholm, 1983; Sebanz and Shiffrar, 2009). Perhaps even more relevant for the embodied simulation argument is that based on kinematics alone human observers can also accurately identify emotions like happiness and sadness (Dittrich et al., 1996; Atkinson et al., 2004) or discern expression intensities in dance movements (Sevdalis and Keller, 2012). Intriguingly, in a recent study by Sevdalis and Keller (2012) judgment accuracy was positively correlated with self-reported empathy indices. This underscores the role of emotions such as empathic feelings for judgments of observed movements and links directly to the convincing evidence that embodied simulation also accounts for how humans empathize with each other (Gallese, 2001; Gallese et al., 2004; de Vignemont and Singer, 2006).

To summarize, because (a) movements convey critical information for action recognition and the inference of cognitive and emotional states of human agents, and because (b) human observers are able to identify action intentions as well as cognitive and emotional states from others' bodily actions by means of embodied simulation, it seems imperative to explore the joint contributions of perceptual, motor, cognitive and emotional processes to deception detection, with the aim to develop an integrative embodied theory of deception detection. A promising way to do so is based on the following observation: if cognitions and emotions influence perception-action-informed judgments about deceptive action intentions, there are essentially two possible ways in which this could be effectuated: (1) either cognitions and emotions influence the perceptual sensitivity directly, which then eventually leads to different judgments, or (2) cognitions and emotions change, that is, shift the judgment criterion (independent of the perceptual sensitivity), thereby causing a judgment bias. Based on some preliminary findings from my own lab (Cañal-Bruland and Schmidt, 2009; Cañal-Bruland et al., 2015), I consider the latter more likely than the former. That is, I hypothesize that cognitions as well as emotions bias the judgments about rather than perception of deceptive actions. However, future research is needed to scrutinize these ideas and thereby improve our understanding of deception detection in action.

## Author contributions

The author confirms being the sole contributor of this work and approved it for publication.

### Conflict of interest statement

The author declares that the research was conducted in the absence of any commercial or financial relationships that could be construed as a potential conflict of interest.
